# Emotional arousal and valence in patients with fibromyalgia: a pilot study

**DOI:** 10.3389/fpain.2023.1075722

**Published:** 2023-05-31

**Authors:** Roberta Ciuffini, Vincenza Cofini, Mario Muselli, Stefano Necozione, Alba Piroli, Alfonso Marrelli

**Affiliations:** ^1^ Clinical Neurophysiology Unit, San Salvatore Hospital, L'Aquila, Italy; ^2^ Department of Life, Health and Environmental Sciences, University of L'Aquila, L’Aquila, Italy

**Keywords:** fibromyalgia, emotional arousal, emotional valence, international affective picture system, central sensitization, social cognition

## Abstract

The pathogenesis of pain in fibromyalgia is still not completely understood. A disrupted emotional modulation could affect the physiology of nociception and contributes to an altered perception of pain. The aim of this study was to test the role of emotional arousal and valence in pain susceptibility in fibromyalgia using the International Affective Picture System (IAPS) paradigm and the Fibromyalgia Severity Scale (FSS). The study focused on comparing emotional arousal and valence between patients with fibromyalgia and the control group. The secondary objective was to examine the correlation between emotional indices and scores on the FSS and the duration of the disease. The 20 patients with fibromyalgia enrolled showed a higher mean arousal score for all the stimuli, including a higher score for unpleasant and socially unpleasant stimuli. The valence scores for social-relevant stimuli were also higher. Increased arousal to unpleasant and socially unpleasant images and increased valence of them correlated with the duration of the disease and the severity of symptoms and could reflect impairment in social cognition and marked sensitivity to pain in interaction with central nociceptive dysregulation.

## Introduction

Relevant advances have been made to better understand the pathophysiology of fibromyalgia (FM) (1) and to propose new treatments based on clinical and preclinical studies (2).

A complex interaction between environmental, psychological, and sensory factors can modulate individual vulnerability to chronic pain. The relationship between an impaired pain regulatory system, sensory inputs, environment, psychological vulnerability, and comorbid psychological conditions can result in individual or multiple functional pain syndromes (3). Data seem to suggest that both central and peripheral mechanisms might be involved in nociceptive system dysfunction in FM (4). FMRI studies have found regional gray matter alterations, reduced functional connectivity of the descending pain modulation system, and increased pain matrix activity (5). Additionally, impaired functional connectivity of the periaqueductal gray (PAG), increased response of the insula, operculum, and midfrontal cortical regions, and decreased response of the lateral frontal cortical regions due to related nociceptive stimulation have been reported as a hypothetical dysfunction of the central system responsible for nociceptive sensory integration (6, 7).

Neurophysiological studies have demonstrated the lack of habituation of the cortical responses and an imbalance of the cortical excitability due to repetitive pain stimuli compatible with the central sensitization mechanism having a possible role (8). Sensitization is a condition that can develop and maintain neuropathic pain involving the brain pain matrix (9) in which neurophysiological changes may be as important as psychological, behavioral, and environmental mechanisms (10). Some clinical symptoms such as pain, allodynia, and hyperalgesia may be caused by the increased sensitivity of the central nervous system to pain processing and transmission (11). Persistent anomalous temporal summation of pain and nociceptive input from peripheral tissues can result in central sensitization. In fact, the International Association for the Study of Pain (IASP) has defined central sensitization as “increased responsiveness of nociceptive neurons in the Central Nervous System (CNS) to their normal or subthreshold input” (12). Woolf (13) proposed defining central sensitization as “an amplification of neural signaling within the CNS that results in hypersensitivity to pain”.

Some researchers have correlated hypersensitivity to pain with impaired central pain modulation (14). Others have found that even emotions can influence the perception of pain (15).

Symptoms such as fatigue, sleep disturbances, cognitive impairment, depression, anxiety, and eating disorders reinforce the hypothesis of psychological involvement in the syndrome, both in cognitive and emotional processes (16). Furthermore, cognitive impairment in several domains has been found in patients with FM (17). A meta-analysis (18) found objective neuropsychological measures regarding the deficit of inhibitory control and of short-term and long-term memory in this syndrome. The same objective cognitive deficits such as forgetfulness, distractibility, and word-finding difficulties were found by Teodoro et al. (19) in patients with FM.

Duschek et al. (20) found that attentional bias towards negative information plays a significant role in the interaction between affective state and increased pain perception while Robin et al. (21) reported that patients with FM show increased recognition of both positive and negative stimuli.

A disruption of the emotional modulation of nociception can contribute to FM (22). Therefore, greater emotional intelligence allows for the pain to be perceived as less intense, and less unpleasant negative emotions can increase or amplify the pain. Patients with FM have deficits in affective processing but this dysregulation can be attributed to defensive action (23). Furthermore, patients with FM are less efficient in modulating pain through the positive effects and may derive fewer benefits from pleasant events (24). Kamping et al. (25) suggest that pain-related information, even when perceived unconsciously, is able to enhance exogenous (automatic) attention, by increasing the neural activity involvement (26).

FM also appears to be associated with a disruption of supra-spinal processes related to positive affect and emotional modulation of pain, but not with circuits from the brain to the spinal cord that modulate spinal nociceptive processes (15).

Studies with event-related evoked potentials have also been reported in the literature showing abnormalities (smaller P2 and Late Positive Potentials and greater N250 amplitude) in patients with FM compared to controls with respect to representative images of angry, painful, happy, and neutral stimuli, suggesting an altered emotional influence on image processing (27).

The emotional dimension deals with the unpleasantness related to the noxious stimuli and with the consequent behavior.

The distinction between the sensory discriminant and the affective component of pain is well-known. The localization of a noxious stimulus requires the activation of the primary somatosensory cortex while the attribution of unpleasantness involves the medial pain system (28). Central sensitization primarily mediates the medial pain system, playing a crucial role in improving the pain response (hyperalgesia, defined as abnormally heightened pain sensitivity) and decreasing the pain threshold (allodynia, a condition in which stimuli cause pain that do not normally) or increasing spontaneous pain. A neurobiological link between pain and emotional states can also be hypothesized. Anticipatory anxiety, fear, and expectation of pain increase neural activity in the anterior cingulate cortex, while positive emotional states reduce it instead (29).

Both impaired regulation of one's own affective emotions and the recognition of those of others have been reported in FM and linked to social cognition impairment.

The role of stress in emotional recognition has been debated (30). Corbett, Weimberg, and Duarte (31) noted that stressed people react more to highly negative images than to negative or neutral images (minimally exciting images), and it is for this reason that patients with FM are characterized by impaired responses of the autonomic nervous system (32).

Emotional reactivity as an expression of the limbic network system can strongly influence autonomic balance, such that patients have shown increased sympathetic activity and reduced autonomic response to stress (33).

The relationship between pain and negative emotions needs experimental studies to clarify the impact of these variables on the patient's behavior functions (34, 35). We studied emotional arousal (EA) and emotional valence (EV) in a sample of patients with FM to understand whether emotional problems could be related to pain perception and how they could interfere with the symptoms of the syndrome.

The rationale of this study is to evaluate the role of EA and EV for emotional patterns in FM to better characterize the alteration of emotional processing mechanisms both in qualitative and quantitative terms and to relate the alterations to the clinical pattern of the syndrome.

We hypothesized that one's emotional state in response to external stimuli, interacting with central sensitization, is related to FM severity and duration.

Even if other measures of emotional processing have been used (36), the International Affective Picture System (IAPS) allows the measuring of EA and EV to visual stimuli with different content so it can measure both general higher arousal scores and elevated activation levels to stimuli regarding their emotional or social content.

The objectives were to compare EA and EV between FM patients and a control group and to investigate the correlation between IAPS indexes (37) and the clinical features of FM.

## Methods

This was an observational study carried out on outpatients of the S. Salvatore Hospital of L'Aquila, in central Italy.

The Ethical Committee of L'Aquila (Italy) approved the study, and it has been performed in accordance with the ethical standards laid down in the 1964 Declaration of Helsinki and its later amendments. We obtained written informed consent from all participants.

### Participants

In total, 20 patients (16 females and 4 males) with FM syndrome (FM group) were recruited for the study among the outpatients from the Clinical Neurophysiology Unit during a six-month period considering their completed eligibility. We excluded from the study all patients with a history of psychiatric symptoms previous or coexisting with the onset of FM syndrome and with an inability to provide informed consent.

Furthermore, 20 healthy subjects (12 females and 8 males) with no clinical evidence or family history of the FM syndrome matched for sex, age, and race-ethnicity were recruited for the control group (Table 1).

**Table 1:** Participants’ characteristics.

	FM patients (20)	Healthy control subjects (20)	*p*-value
Age (mean ± sd/median IQR)	36.2 ± 8.4	35.0 ± 5.5	*t* = −0.5,362
	(38.5; IQR:13.5)	(36.0; IQR:9.0)	*p* = 0.5,949^a^
Educational background			
Primary school	2	2	Chi = 0.1,309
High school	13	12	*p* = 0.937^b^
Graduate school	5	6	
Marital status			
Married	12	13	Chi = 0.8,178
Never married	4	5	*p* = 0.664^b^
Divorced/Widowed	4	2	
Occupation			
Employed	12	15	Chi = 1.0,256
Unemployed	8	5	*p* = 0.311^b^
Income per year			
Low	7	3	Chi = 2.4,333
Medium	11	13	*p* = 0.296^b^
High	2	4	
BDI-II (mean ± sd)	13.0 ± 1.8	12.1 ± 1.5	*t* = −1.8,077
			*p* = 0.0,786^a^
SAS (mean ± sd)	20.1 ± 3.1	19.2 ± 3.3	*t* = −0.8,410
			*p* = 0.4,056

IQR, interquartile range.

^a^ Student's *t* test.

^b^ Chi-square test.

The diagnosis of FM was made by a senior neurologist and defined according to the American College of Rheumatology (ACR) (38) including criteria such as chronic widespread musculoskeletal pain, morning stiffness, insomnia, fatigue, cognitive problems, and, often, depression and headache. In addition to these complaints, and due to the main interest in pain symptoms, tender points was another criterion. The recruited patients with FM typically had a minimum of 11 tender points (of a total of 18 specific tender points), which are characterized by decreased pressure pain thresholds that result in hyperalgesia and/or allodynia. No patient**s** were taking medications.

### Clinical assessment

Clinical Global Impression-Severity (CGI-S) (39) was used to evaluate behavioral disturbances by a senior neurologist (AM) based on forty years of clinical experience. The Clinical Global Impression (CGI) was also used to evaluate a nuclear symptom *a priori* defined as emotional involvement and reaction to the pathological condition.

This experience formed the basis of CGI-S of illness rating along a 7-point Likert scale where 1 is “normal, not ill” and 7 is “extremely ill”.

**Figure 1:**
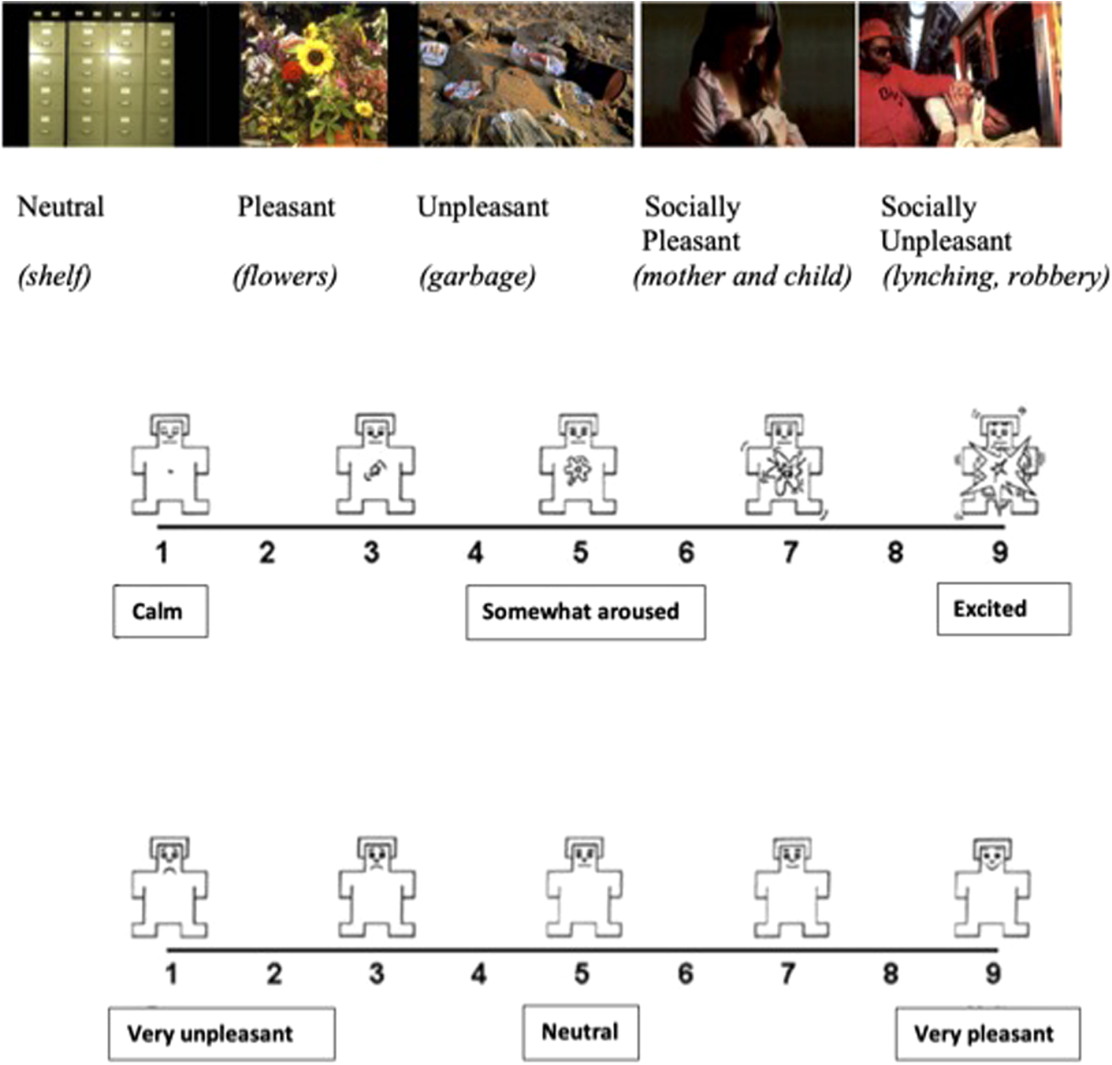
Cartoon-type pictures for evaluating arousal and valence from IAPS.

EA and EV assessments were carried out using the IAPS paradigm which consists of a set of static images based on a dimensional model of emotion. In total, 90 color pictures were chosen from IAPS depicting events with different kinds of affective valence, i.e., unpleasant, pleasant, and neutral events. Unpleasant and pleasant events are also distinguished in these pictures as involving or not involving social human conditions. For example, pictures with social involvement included depictions of mother–child or familial interactions (pleasant) and outcomes of violence (unpleasant) while pictures without social involvement included landscape scenes or flowers (pleasant) and snakes, contamination, or pollution (unpleasant). Neutral images consisted of pictures of furniture or appliances. The five emotional categories of the IAPS are: (1) neutral, (2) pleasant, (3) unpleasant, (4) socially pleasant, and (5) socially unpleasant (Figure 1). For each of the five categories, 18 images were randomly shown using the appropriate software. Reactivity to the pictures was rated based on EA and EV. The valence rating instructions were “Rate how unpleasant or pleasant the image makes you feel using a 1–9 valence scale (1 = very unpleasant, 5 = neutral, 9 = very pleasant)”. The arousal rating instructions were “Rate how emotionally intense or arousing the image makes you feel using a 1–9 scale arousal scale (1 = calm, 5 = somewhat aroused, 9 = excited)”. The valence scale consisted of a cartoon-type figure in which nine human emotional expressions, ranging from smiling and happy to frowning and unhappy, were presented. The arousal scale consisted of another cartoon-type figure with nine expressions ranging from calm and relaxed to excited and wide-eyed. Stimuli presentation and response recording were managed using custom software (Super Lab 4.0 for Windows).

Figure 1 shows the cartoon-type pictures used for evaluating arousal and valence.

The subjects were tested individually in a dimly lit room. They were seated in front of a 15-inch computer monitor at 50 cm. During the test session, subjects were instructed that a series of 90 trials would be presented and that for each trial they would be asked to rate the valence and arousal of that picture.

Trials started with a 2-s full-screen presentation of one picture. The presentation order of the pictures depicting neutral, pleasant, and unpleasant scenes, with or without social involvement, was randomized for each subject. Then, after a 1-s black screen, a display containing a smaller version of the same picture (located in the upper part) and the valence scale (located in the lower part) was presented. This display remained visible for 3 s or until the participant responded. After the participants' valence rating, another display was presented in which the valence scale was substituted with the arousal scale. Similarly, the display remained visible for 3 s or until the participant responded. If the subjects did not respond within 3 s, an omission was recorded. Those who showed more than 5% of omitted responses were excluded from the two samples and were not considered in the analysis. No subjects in our study were excluded for this reason.

To evaluate the clinical features of FM, the duration of the illness (months) was noted and the Fibromyalgia Severity Scale (FSS), consisting of both the Widespread Pain Index (WPI) and Symptom Severity Scale (SSS) (40), was administered to the patients by the same neurologist (AM).

All the patients underwent an anxiety and depression evaluation by means of the Zung Self-Rating Anxiety Scale (SAS) and Beck Depression Inventory-II (BDI-II).

The SAS is a 20-item self-report rating scale built to measure anxiety levels, based on scoring in 4 groups of symptoms: cognitive, autonomic, motor, and central nervous system symptoms. Each question is scored on a Likert-type scale of 1-4 (based on these replies: “a little of the time,” “some of the time,” “good part of the time,” “most of the time”). The total scores range from 20 to 80. The standardized cutoffs are 21-40: low anxiety level; 41-60: moderate anxiety level; and 61-80: high anxiety level (41).

The BDI II is a psychometric test for measuring the severity of depression. It contains 21 questions; each answer being scored on a scale value of 0 to 3. Higher total scores indicate more severe depressive symptoms. The standardized cutoffs are 0–13: minimal depression, 14–19: mild depression, 20–28: moderate depression, and 29–63: severe depression (42).

The patients with SAS scores higher than 40 and/or BDI scores higher than 20 were excluded.

The patients enrolled in the study showed BDI II scores equal to or lower than 16, and SAS scores not exceeding the value of 25.

### Statistical analysis

This was a convenience sample, and a formal sample size calculation was not performed.

Continuous data were initially tested for equality of variances using Levene's test, and the Shapiro normality test was used subsequently to test for normality. Based on these results, in case of homogeneity of variances and normal distribution of data, the statistical comparison was performed using either Student's t-test or the Mann–Whitney U-test.

Categorical variables were compared using the chi-square test; in the cases where the expected frequencies were less than 5, the Fisher test was used.

Correlation analyses among the patients with FM between IAPS, duration of illness, and FSS scores were performed using the nonparametric Spearman rho coefficient. The Bonferroni method was used to adjust multiple comparisons. A *p*-value of <0.05 was considered statistically significant. All analyses were performed using STATA 14/MP software.

## Results

The study was not sufficiently powered. It was a pilot study to explore data.

In total, 40 subjects of Caucasian ethnicity, distributed equally in the FM and control groups, participated in the study. The average CGI score recorded was 2.2 for the FM group and 1.9 for the control group and no statistically significant difference was appreciated.

Student's *t*-test showed that there were no significant differences between the FM group and the control group with respect to depression [13.0 ± 1.8 vs. 12.1 ± 1.5 (*t* = −1.8077; *p* = 0.0786)] and anxiety [20.1 ± 3.0 vs. 19.2 ± 3.3 (*t* = −0.8410; *p* = 0.4056)] The interquartile range is specified in Table 1.

The investigated groups had similar social and demographic characteristics (*p* > 0,05) as reported in Table 1.

Analyzing the IAPS scores, the arousal ratings were statistically different between the FM group and the control group (*p* < 0,05) for all the pictures**.** In particular, the FM group had a higher mean score in every type of picture shown (neutral, unpleasant, pleasant, socially unpleasant, and socially pleasant). However, the valence rating was different between groups for socially pleasant pictures (*p* = 0.0044) and for socially unpleasant pictures (*p* = 0.0485) with the FM group showing a significantly higher score (Table 2).

**Table 2:** Comparison between FM group and control group of Pictures arousal and valence scores.

	neutral	unpleasant	pleasant	socially unpleasant	socially pleasant
Arousal					
FM group	3,1 ± 0,5	6,**9**8 ± 0,4	6,**2**1 ± 0,3	7,0 ± 0,4	6,5 ± 0,5
Control group	2,6 ± 0,4	6,4 ± 0,3	5,9 ± 0,4	6,2 ± 0,75	5,69 ± 1,10
**Test result**	**−3.636**	**−4.0530**	**−2.2558**	**−3.750**	**−2.801**
**p-value**	**0.0008** ^1^	**0.0002** ^1^	**0.0299** ^1^	**0.0001** ^2^	**0.0017** ^2^
Valence					
FM group	2,87 ± 0,5	3,2 ± 1,4	5,9 ± 0,7	2,0 ± 0,6	6,3 ± 0,6
Control group	2,93 ± 0,6	3,4 ± 0,9	5,7 ± 1,0 ,4	2,40 ± 0,4	5,6 ± 1,0
**Test result**	**0.9185**	**0.6866**	**−0.6522**	**2.0382**	**−3.186**
**p-value**	**0.3641** ^1^	**0.4965** ^1^	**0.5182** ^1^	**0.0485** ^1^	**0.0044** ^2^

Bold values indicated the significant ones.

^1^ Student's t test.

^2^ Mann-Whitney U-test

The arousal scores of unpleasant and socially unpleasant pictures were highly positively related to the duration of illness and to the SSS score (Table 3).

**Table 3:** Spearman correlation matrix in patients with fibromyalgia.

		Duration of illness	WPI	SSS	Neutral arousal	Neutral valence	Unpleasant arousal	Unpleasant valence	Pleasant arousal	Pleasant valence	Socially unpleasant arousal	Socially unpleasant valence	Socially pleasant arousal	Socially pleasant valence
WPI	**rho**	0,42	1											
* *	*p-value*	*0,06*	* *	* *	* *	* *	* *	* *	* *	* *	* *	* *	* *	* *
SSS	**rho**	0,72	0,50	1										
* *	*p-value*	*0,00*	*0,03*	* *	* *	* *	* *	* *	* *	* *	* *	* *	* *	* *
Neutral														
Arousal	**rho**	0,02	0,30	0,13	1									
* *	*p-value*	*0,93*	*0,19*	*0,58*	* *	* *	* *	* *	* *	* *	* *	* *	* *	* *
Neutral														
Valence	**rho**	0,10	0,25	0,26	0,28	1								
* *	*p-value*	*0,68*	*0,29*	*0,26*	*0,23*	* *	* *	* *	* *	* *	* *	* *	* *	* *
Unpleasant														
Arousal	**rho**	0,58	0,07	0,56	−0,05	0,29	1							
* *	*p-value*	*0,01*	*0,78*	*0,01*	*0,84*	*0,22*	* *	* *	* *	* *	* *	* *	* *	* *
Unpleasant														
Valence	**rho**	0,13	0,04	0,10	0,06	−0,07	0,17	1						
* *	*p-value*	*0,59*	*0,88*	*0,68*	*0,81*	*0,76*	*0,48*	* *	* *	* *	* *	* *	* *	* *
Pleasant														
Arousal	**rho**	0,01	0,04	−0,06	0,46	0,02	−0,07	−0,28	1					
* *	*p-value*	*0,97*	*0,86*	*0,79*	*0,04*	*0,93*	*0,76*	*0,23*	* *	* *	* *	* *	* *	* *
Pleasant														
Valence	**rho**	0,07	0,18	0,34	−0,16	−0,42	−0,01	0,25	−0,09	1				
* *	*p-value*	*0,76*	*0,45*	*0,15*	*0,51*	*0,06*	*0,97*	*0,29*	*0,71*	* *	* *	* *	* *	* *
Socially														
Unpleasant														
Arousal	**rho**	0,71	0,26	0,66	−0,18	0,00	0,46	−0,07	0,04	0,33	1			
* *	*p-value*	*0,00*	*0,28*	*0,00*	*0,45*	*0,98*	*0,04*	*0,78*	*0,88*	*0,16*	* *	* *	* *	* *
Socially														
Unpleasant Valence	**rho**	0,08	−0,20	−0,04	−0,45	−0,09	0,38	0,64	−0,48	0,06	−0,02	1		
* *	*p-value*	*0,75*	*0,39*	*0,86*	*0,05*	*0,69*	*0,10*	*0,00*	*0,03*	*0,80*	*0,93*	* *	* *	* *
Socially														
Pleasant														
Arousal	**rho**	−0,01	0,03	0,13	0,03	−0,22	0,21	0,19	−0,17	0,23	−0,07	0,26	1	
* *	*p-value*	*0,96*	*0,91*	*0,59*	*0,91*	*0,35*	*0,36*	*0,43*	*0,48*	*0,32*	*0,76*	*0,28*	* *	* *
Socially														
Pleasant														
Valence	**rho**	0,24	0,31	0,14	0,26	−0,17	−0,02	−0,04	0,39	0,23	−0,03	−0,33	0,30	1
* *	*p-value*	*0,30*	*0,18*	*0,55*	*0,27*	*0,47*	*0,93*	*0,86*	*0,09*	*0,33*	*0,91*	*0,15*	*0,19*	* *

In total, 12 patients (60%) had BDI-II scores between 10 and 13 (minimal depression) and 8 patients (40%) between 14 and 16 (mild depression). Furthermore, 12 patients (60%) had SAS scores between 14 and 20 (very low anxiety level) and 8 patients (40%) between 22 and 25 (low anxiety level). The scores for depression and anxiety did not support a relevant clinical diagnosis. Due to the clinical features of FM, it is common to find some psychological symptoms, but, if these do not sustain a well-defined DSM-V disorder, we considered the patients with FM to be without psychiatric comorbidity. The even smaller sample of patients with some depressive and/or anxious responses to the test does not allow for a reliable statistical analysis.

## Discussion

The main objective of this study was to compare emotional arousal and valence between patients with fibromyalgia and a control group, specifically examining the role of emotional arousal and valence in these patients' pain susceptibility, through the paradigm of the IAPS and the FSS. The secondary purpose of the study itself was to investigate the correlation between emotional indices, FSS scores, and disease duration.

The intensity of negative emotions is positively associated with increased pain intensity, irritability, physical and mental tension, functional limitations, number of sensitive points, insomnia, cognitive deficits, fatigue, and the impact of the disease on quality of life. These patients often feel isolated, misunderstood, or rejected by relatives, friends, health professionals, and in general by their social context.

Successful adaptation to chronic pain thus requires the ability to self-regulate or exercise control over one's bodily symptoms, thoughts, emotions, and behaviors (43). In particular, emotion regulation has been found to be critical in adaptation to chronic pain (44).

Koechlin et al. (3) suggest that this link may be because failed emotion regulation may maintain or even worsen pain and limit the person's overall functioning. This can in turn feed back into one's level of affective instability and, as such, become a vicious circle of reinforcement. However, it may also be that persistent pain and emotion dysregulation share similar underlying mechanisms (45). For example, negative repetitive thinking could operate as a transdiagnostic factor, that is, serve as a driver for emotional and pain-related problems (45, 46). When this pattern of repetitive thinking becomes an ineffective form of problem-solving, it drives the development of emotional and physical problems (45, 47, 48).

Specifically, the stress associated with the experience of chronic pain reduces glutamate, an excitatory neurotransmitter in the medial prefrontal cortex, resulting in emotional dysregulation.

Collectively, these results demonstrate that neurobiological processes underlie emotion dysregulation in chronic pain.

Chronic pain presents constant challenges to the person with FM and requires adequate coping strategies. Due to these constant challenges, it is likely that in some cases the flexible adaptation of coping strategies to the context will fail or that, after continuous challenges to deal with chronic pain and/or related problems, the individual will not be able to cope with these challenges, resulting in the variability of negative emotions (43).

Many pain syndromes such as migraine (49), lower back pain, dysmenorrhea, temporomandibular dysfunction, irritable bowel syndrome, and FM can be partially explained based on the central sensitization mechanism. Chronic pain caused by central sensitization can be maintained by a persistent peripheral input, even if minimal. Many additional factors can contribute to the chronicity of the pain (50). It has been described that people who develop FM have a specific type of personality (51). Patients with FM could be more conscientious and agreeable than the wider population. Anxiety is a relatively constant feature and depressive symptoms have been found (52).

Neuroimaging and neurophysiological approaches have brought experimental evidence to support the network disruption, but they do not show clinical usefulness to detect and quantify cognitive and behavioral disturbances in pain syndromes. A neuropsychiatric approach and a neuropsychological assessment represent important diagnostic tools (53, 54).

Stimulation by images with different affective content affects subjective pain for a cognitive mechanism of attentive engagement (55).

Godinho et al. (56) showed that the intensity of the pain significantly increased when painful stimuli were concomitant to images showing human pain, whereas pictures with identical emotional values but without somatic content failed to modulate pain.

An abnormal sustained arousal or sustained “stress” response to environmental stimuli may facilitate the establishment of a low threshold for pain and a central sensitization state, contributing to the development and maintenance of pain syndromes (57). Cognitive components of pain susceptibility and an emotional predisposition to a lower pain threshold which are linked to the decreased input of peripheral stimuli and central sensitization could support a multidimensional pathogenesis of the syndrome and explain the heterogeneity of the phenotype expression due to the relative contribution of central and peripheral components in different individuals (58).

Our results seem to confirm the hypothesis that EA and EV can be considered involved in the clinical expression of FM. In our sampling, arousal was increased in response to the pictures of the IAPS and increased more significantly to unpleasant stimuli and pictures with social relevance. Valence seemed to be higher for the stimuli with social context. From a speculative point of view, this could underline the role of social cognition as a psycho-biological terrain of the increased susceptibility to pain perception in FM. Not only is the regulation of one's own emotions altered in FM but also, even less referred to, the detection of emotional signals from the environment (36) and representation of other people's mental states is altered too. Social cognitive skills can be altered, and the Theory of Mind (ToM) must be considered to be involved in FM (36). Pain can depend on the emotional context and the individual's psychological state. Emotional and cognitive factors influence the way every individual feels pain (9) including hyperalgesia and allodynia, which are related to central sensitization. One's attentional state, pleasant and unpleasant emotions, and empathy can affect the activity of afferent pain pathways (8). The affective component is relevant to the pain experience and to adequate behavior.

The anterior cingulate cortex and the insula are involved in the emotional aspect of pain and the link between pain threshold and emotional state could be established in the limbic system (59). Abnormal arousal may have a reasonable relationship with the mechanism of central sensitization, acting as a stressor (60).

Otherwise, central sensitization itself can induce greater pain which acts as a stressor, driving abnormal emotional reactivity. The arousal scores of unpleasant and socially unpleasant pictures were highly positively related to the duration of illness and to the SSS score.

The higher arousal elicited by neutral stimuli shows some kind of modality-unspecific dispositional baseline of emotional arousal (61). Emotional awareness is probably a trait aspect of emotional regulation that influences emotional arousal in FM. Nevertheless, the patients with FM showed an even more elevated activation in response to negative stimuli and pictures with social content.

A reliable link between abnormal arousal and stress may reside in individual efficiency in terms of emotional intelligence. If the latter is high, it acts as a stress buffer. If it is low, exposure to stressful situations causes less adaptive responses, insufficient coping strategies, and stress (62). Stressors are likely to be one of the main risk factors for FM (63).

The patients with FM showed different patterns of arousal during the observation of the images. Due to the lesser adaptive capacity to all stimuli, they manifested abnormal arousal to all the images regardless of the content, including emotionally neutral ones (26). Furthermore, due to the social cognition alteration, the higher arousal was even more evident for images with pleasant or unpleasant situations involving people.

EV impairment seems to be related to the duration of the illness and to the severity but not to the distribution of pain (64). Chronic pain can, reversely, affect the pain-modulating system, exacerbating pain perception.

On these bases, emotional pathology could represent both the prerequisite and the consequence of pain. The correlation with the duration of illness indicates the possibility of the establishment of a vicious circle between alteration of the ER and the syndrome that, over time, causes a further emotional dysfunction linked to the factors of chronic pain and amplification, regardless of the presence of anxiety and depression (65).

An alternative interpretation could be that both pain sensitivity and emotional reactivity are related to dysfunction in the limbic system. Localization of the pain may depend more on peripheral factors. Patients with FM report high levels of disease burden in terms of pain and health status (66).

Moreover, there is evidence that the decoding of emotions in patients with FM is impaired, regardless of psychological comorbidities and drugs.

In our study, the effects of psychological symptoms such as anxiety and depression are relatively negligible since patients with SAS scores higher than 40 and/or BDI scores higher than 20 were excluded**.**

Based on these considerations, many studies using evidence-based psychological therapies have been made for FM (67). Group treatment with a cognitive-behavioral approach, intensive and remote treatments, multimodal therapy, hypnosis, behavioral therapies, mind-body-based techniques (68), and biofeedback are often used. Emotional awareness and expression therapy have been used to achieve a reduction in widespread pain (29). A meta-analysis (69) to assess the effectiveness of psychological treatment for FM across five different outcomes, namely pain, sleep, depression, catastrophizing, and functional status, showed that this intervention for FM can be comparable to the effects of drug treatment and more favorable than other non-psychological treatment.

FM can be considered a heterogenous condition. In its pathogenesis, peripheral and centrally driven painful conditions coexist. Patients fall along a continuum in which both impairments may be predominant (70). Brain functions have a crucial role in perceiving pain (71), and the assessment of cognition as part of an integrative biopsychosocial model, including emotional status and traits, could represent a key point in the diagnosis and treatment of the disorder. Our results seem to underline the role of altered emotional processing in the pathogenesis of FM and the correlation with pain perception, the clinical features of the syndrome, and the duration of illness.

## Limitations of the study

The study has several limitations. The main one is that the clinical sample is small, due also to the situation determined by the COVID-19 pandemic. It is not easy to increase the sample size, but it is useful to continue the research as soon as possible depending on the course of the pandemic. Moreover, the tests must be administered in the presence of the patients because the images of the IAPS must be presented under the supervision of a psychologist.

No causal inference is demonstrable based on the study design but some relationships among the different issues can be hypothesized. Moreover, there was no assessment using the IAPS preceding the onset of FM.

Future studies are needed to better clarify the role of emotional testing in patients with FM, taking into account the differences between the various measuring instruments adopted. With adequate weighting, IAPS could be adopted as an additional tool to evaluate emotional arousal and valence. Moreover, the relationship between a general greater emotional excitability and reactivity to stimuli with social content must be focused on.

## Data Availability

The raw data supporting the conclusions of this article will be made available by the authors, without undue reservation.
